# A behavioural approach to key area identification in seabirds for threat mitigation and spatial management

**DOI:** 10.1186/s40317-025-00427-z

**Published:** 2025-10-21

**Authors:** Hannah Wood, Emma J. Tebbs, Robin Freeman, Mark Bolton, Ian R. Cleasby, Francis Daunt, Jonathan A. Green, Mark A. Newell, Stephen F. Newton, Ellie Owen, Alice M. Trevail, Catharine Horswill

**Affiliations:** 1 Department of Geography, King’s College London, Bush House, North East Wing, 40 Aldwych, London, WC2B 4BG UK; 2 Institute of Zoology, Zoological Society of London, Regents Park, London, NW1 4RY UK; 3 RSPB Centre for Conservation Science, The Royal Society for the Protection of Birds, The Lodge, Sandy, Bedfordshire, SG19 2DL UK; 4 RSPB Centre for Conservation Science, North Scotland Regional Office, Inverness, IV2 3BW UK; 5 UK Centre for Ecology & Hydrology, Bush Estate, Penicuik, Midlothian EH26 0QB UK; 6 School of Environmental Sciences, University of Liverpool, Liverpool, L69 3GP UK; 7 BirdWatch Ireland, 20D Bullford Business Campus, Kilcoole, County Wicklow, A63 RW83 Ireland; 8 National Trust for Scotland, Balnain House, 40 Huntly Street, Inverness, IV3 5HR UK; 9 Environment and Sustainability Institute, University of Exeter, Penryn, UK; 10 Centre for Biodiversity and Environmental Research, Department of Genetics, Evolution and Environment, University College London, London, UK

**Keywords:** Biotelemetry, Animal distribution, Kernel density estimates, Hidden Markov model, Conservation planning

## Abstract

**Background:**

Identifying key areas of animal distribution using individual movement data is fundamental for conservation planning, threat mitigation, and spatial management. Methodologies which define these areas based on measures of high density and abundance may overlook spatial heterogeneity in behaviour-specific distributions. This is particularly relevant for behaviours that occur at lower densities but are associated with increased exposure to specific environmental threats. We used a dataset of 566 GPS tracked individuals and 14 colonies of a vulnerable species of seabird, the black-legged kittiwake (*Rissa tridactyla*), to compare two methods for delineating key areas. The first method applies kernel density estimates, based on 50% (‘core area’) utilisation distributions, to all movement data during an at-sea trip. This reflects a widely used density-based approach to identify high-use spatial areas. The second method incorporates hidden Markov modelling to classify movement data into three dominant behaviour states: resting, foraging, and transiting, to identify behaviour-specific high-use areas. We then compare population-level estimates of key areas based on each method using the BirdLife International Key Biodiversity Area framework. We also explore how the selection of an intermediate (70%) and home range (95%) utilisation distribution influences the capture of different behaviours.

**Results:**

We found that individual-level kernel density estimates based on core areas of all movement data fail to adequately capture the core distribution of transiting, a widespread and dispersed behaviour. Moreover, population-level estimates of key areas derived from transiting behaviour are significantly larger than those identified using all tracking data, suggesting that conventional methods likely underestimate exposure to threats encountered during transit. Conversely, key areas for resting and foraging behaviour are more spatially constrained than those derived from all movement data, implying that behaviour-specific analyses may improve the precision of conservation planning. Both individual and population-level key area estimates based on larger utilisation distributions (i.e. 75% and 95%) better capture the distribution of transiting behaviour as these larger distributions probabilistically encompass a greater fraction of observed movement trajectories.

**Conclusion:**

These results highlight the importance of labelling movement data by behavioural state to enhance the utility of GPS data for conservation applications. By incorporating behavioural state differentiation into spatial analyses, threat exposure assessments can be refined to focus conservation resources more effectively. Furthermore, this approach has direct implications for environmental impact assessments, particularly in the context of expanding marine industries such as offshore renewable energy developments.

**Supplementary Information:**

The online version contains supplementary material available at 10.1186/s40317-025-00427-z.

## Background

Marine biodiversity is under growing pressure from the cumulative effects of anthropogenic activities [5, 16, 23, 59, 69]. One way to mitigate biodiversity loss is the identification and protection of habitats critical to species persistence. For marine spatial planning, including the designation of protected areas, to be effective, it must balance conservation with sustainable development and secure support from all affected stakeholders [1, 2, 29, 73, 74, 93]. Overly broad or poorly placed protections risk creating conflict with economic activities, reducing enforcement and compliance, and delivering weak conservation outcomes [57]. By refining the identification of key areas that are critical for species persistence, conservation practitioners can focus protection effort efficiently while minimising unnecessary restrictions on human activities [44].

Global Positioning System (GPS) tracking of free ranging individuals has become a widely used tool for investigating movement and space use, providing critical insights for conservation strategies [17, 27, 62, 79, 84]. Tracking data are often analysed with density-based methods to delineate high-use areas, such as breeding colonies and primary foraging sites [63, 72, 76, 78, 80]. In parallel, Hidden Markov models (HMMs) are widely used in behavioural ecology to classify movement into behavioural states, yet their outputs are rarely incorporated directly into conservation planning frameworks [12, 35, 58, 72, 83, 85, 94]. Areas of high spatial density may not align with regions where individuals are most exposed to environmental risks. Individuals may encounter impacts during periods of lower aggregation, or when exhibiting specific behaviours. For example, analyses of seabird tracking data have identified spatio-temporal overlaps between foraging activity and commercial fishing zones, increasing bycatch risk [26, 19]. Similarly, seabirds may be particularly vulnerable to collision and barrier effects from offshore wind energy developments during transiting flights [38, 52, 104]. Consequently, methods that identify key spatial areas solely based on high density and abundance may underestimate exposure to environmental risks, with implications for conservation effectiveness.

Seabirds are amongst the most threatened avian groups globally, with many species in long-term decline [10, 30, 40]. Seabird conservation planning typically targets high-density use areas, such as Key Biodiversity Areas (KBAs), to inform the designation of protected areas, such as Special Protection Areas (SPAs) and Marine Protected Areas (MPAs) [9, 34, 55, 78]. One common method for delineating high use areas is to use the 50% utilisation distribution, the smallest area expected to contain 50% of all locations, irrespective of behaviour [6, 36, 78]. While the 50% utilisation distribution is effective for delineating high-use areas [80, 90, 103, 116], it can miss behaviours expressed at lower spatial densities, such as transiting, when individuals move directionally between areas used for other higher aggregation behaviours, such as foraging or breeding. These commuting corridors carry high energetic value, with route selection constrained by navigational efficiency, resource distribution and environmental conditions. Barriers created by offshore wind developments can therefore impose energetic costs of avoidance [28, 41, 43, 81, 86, 115].

The proportion of time seabirds spend in different behavioural states may vary between colonies and across different phases of the breeding season, particularly as central place constraints associated with chick provisioning relax [15, 21, 70, 98]. Breeding seabirds may also alter their foraging strategies in response to adverse conditions [21, 46, 66, 71]. Larger utilisation distributions, such as 95%, can provide broader home range estimates [47, 95, 112],  yet may still fail to capture dispersed or infrequent behaviours, or areas of specific threats.

Hidden Markov models (HMMs) classify movement into behavioural states and are widely used in seabird behavioural ecology [12, 35, 58, 83, 85, 94], yet they remain rarely integrated into spatial planning [72]. Incorporating state-specific distributions into key-area identification could improve threat assessments at individual and population levels, highlight energetically efficient commuting corridors, and align management actions with periods of heightened exposure [86, 97, 100, 105].

To test whether behavioural-state classification can refine key area identification we utilised GPS tracking data from black-legged kittiwakes (*Rissa tridactyla*, hereafter kittiwake), a globally widespread and vulnerable species of seabird. Approximately 6% of the global kittiwake population breeds in the UK and Ireland during the boreal summer  [14]. During this period, kittiwakes act as central place foragers, repeatedly returning to nesting sites to incubate eggs and provision offspring [32]. Kittiwakes are of high conservation priority and categorised as Vulnerable on the IUCN Red List having declined by 43% in Britain and Ireland between census periods in 1998–2002 and 2015–2021 [14]. This decline has been primarily attributed to reductions in prey availability driven by overfishing and climate change [20, 32, 33, 37, 50, 60, 89, 106, 114]. The rapid expansion of offshore renewable energy developments also poses a growing threat, with kittiwakes being particularly vulnerable to increased mortality from collisions with turbine blades [42, 49, 52, 96]. Given the cumulative pressures faced by long-lived seabird species, proactive conservation management is required [67, 68].

In this study, we examine how estimates of key spatial areas differ at the individual and population-level when using (1) all at-sea movement data versus (2) tracking data classified into specific behavioural states. We hypothesise that defining key spatial areas based on movement data will underrepresent areas critical for behaviours expressed at lower density and abundance, for example transiting between breeding and foraging grounds. We discuss the implications of this bias for environmental impact assessments and conservation planning.

## Methods

### Tracking data

This study uses individual-based movement data collected from kittiwakes breeding in the UK and Ireland. Movement data were collected between May and July from 2010 to 2015 as part of the FAME (Future of the Atlantic Marine Environment) and STAR (Seabird Tracking and Research) projects, led by the Royal Society for the Protection of Birds (RSPB) (for summary see suppl. info. Appendix 1, Table S1.1). Data were collected from incubating and chick-rearing adult kittiwakes fitted with GPS loggers (i-GotU GT-120, Mobile Action). Loggers were programmed to record locations at 100-s intervals. Devices were affixed to the birds’ back feathers between the wings or to tail feathers using waterproof tape (Tesa SE, Norderstedt, Germany), with total instrument mass below 5% of body mass, or 3% where tail attachments using smaller batteries were used. Additional details of deployment procedures can be found in Wakefield et al., [113] and Trevail et al., [110]. Little evidence of tag effects have been identified by Cleasby et al., [25].

**Fig. 1:**
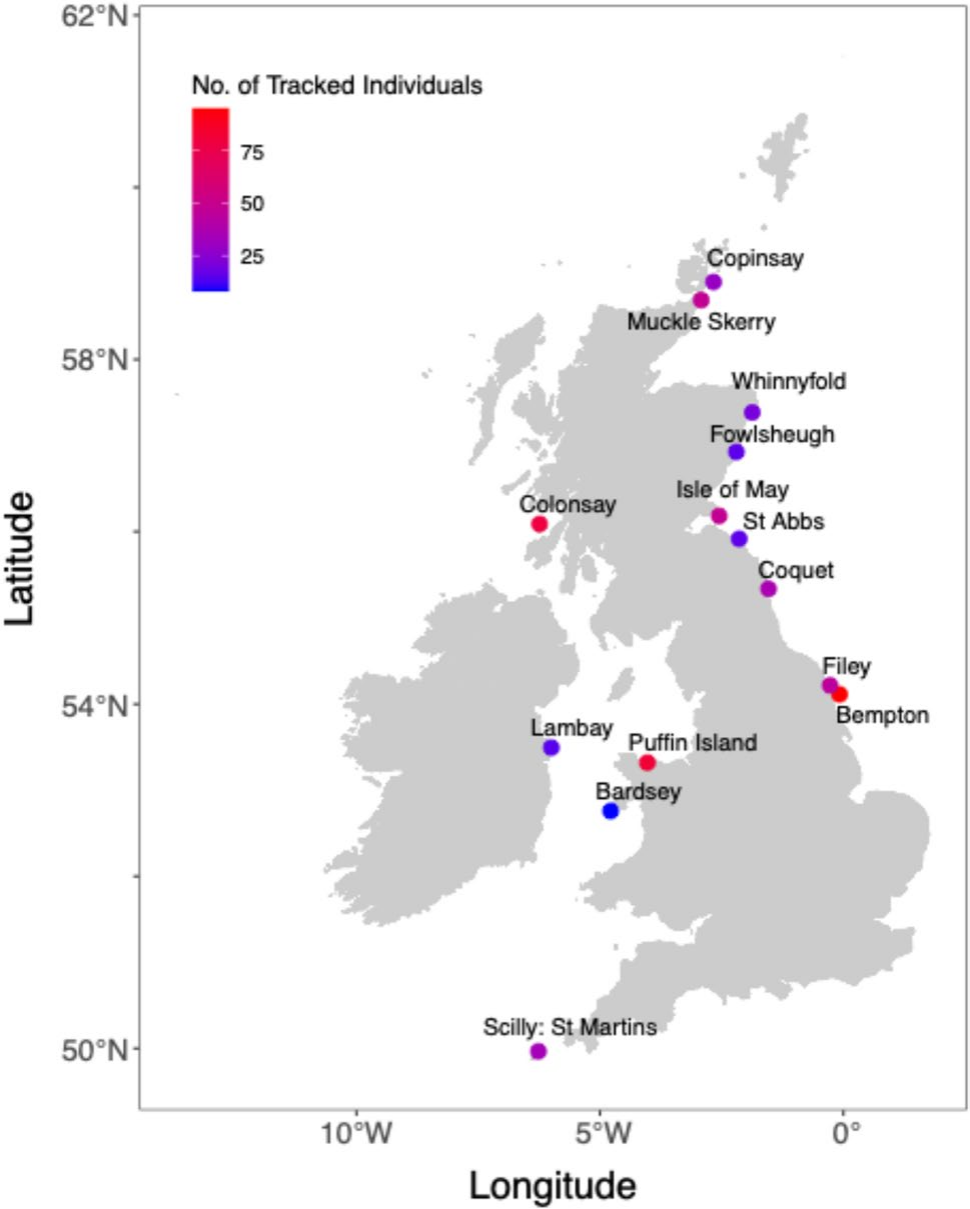
Tracking data used in this study was comprised of 1930 trips from 566 individuals across 14 colonies. Colonies are colour coded by number of tracked individuals

Data analysis was conducted using program R (v. 3.2.2, [101]). Colonies with fewer than eight tagged individuals with complete trips or fewer than three individuals tagged per year were excluded [111], resulting in a final dataset of 566 individuals and 14 colonies (Fig. 1). A breakdown of tracked individuals per site and year is provided in suppl. info. Appendix 1, Table S1.2, and a full workflow for this study is given in suppl. info. Appendix 1, Fig. S1.1. The dataset was further refined by splitting GPS tracks into individual trips, removing stationary location fixes at colonies, and eliminating incomplete trips where birds failed to return to the colony. Trip segmentation was performed using the tripSplit function in the R package Track2KBA (v. 1.0.5, [6]), employing a 500 m inner buffer to define colony locations and a 1 km return buffer with a minimum return duration of 14 min to delineate trips. This method has been validated for removing resting and washing behaviours near the colony [111]. Trip metrics were calculated for each retained colony, including mean and standard deviation of trip distance, maximum trip distance, and trip duration.

### Classifying movement behaviour

To classify movement behaviour, we applied hidden Markov models (HMMs) to the GPS data using the R package moveHMM (v. 1.8, [88]). We selected HMMs due to their ability to infer discrete behavioural states from continuous tracking data while accounting for temporal autocorrelation [61, 83, 102]. Following a previous kittiwake study [110], tracking data were not interpolated and we employed a three-state HMM to classify movement into resting, foraging, and transiting behaviours. Using three-state HMMs also reflects an established understanding of at-sea seabird behaviour and makes our analysis comparable to other seabird studies [11, 21, 22, 35, 92, 99, 109]. We used the Viterbi algorithm to determine the most probable sequence of movement states [88, 117], and defined parameter distributions based on previously established values for kittiwakes [110] with a gamma distribution for step lengths and a von Mises distribution for turning angles, see suppl. info. Appendix 1, Table S1.3.

### Comparing key area estimates using ‘all behaviour’ track data versus separated behaviours

We used a two-step process to compare key areas estimated from total (‘all behaviour’) tracking data with those derived from behaviour-specific data. First, kernel density estimates (KDEs) were generated at the individual level for ‘all behaviour’ and each classified behaviour separately, then the spatial overlap between these estimates was calculated. Second, population-level key spatial areas were estimated and compared for ‘all behaviour’ and each behavioural state using the BirdLife International Key Biodiversity Area framework [6], for infographic of work pipeline (see suppl. info. Appendix 1, Fig. S1.1). KDEs were computed using the Track2KBA package, with a 50% utilisation distribution threshold selected to delineate ‘core areas’ of space use [6, 108, 113].

#### Individual-based kernel density estimates

**Fig. 2:**
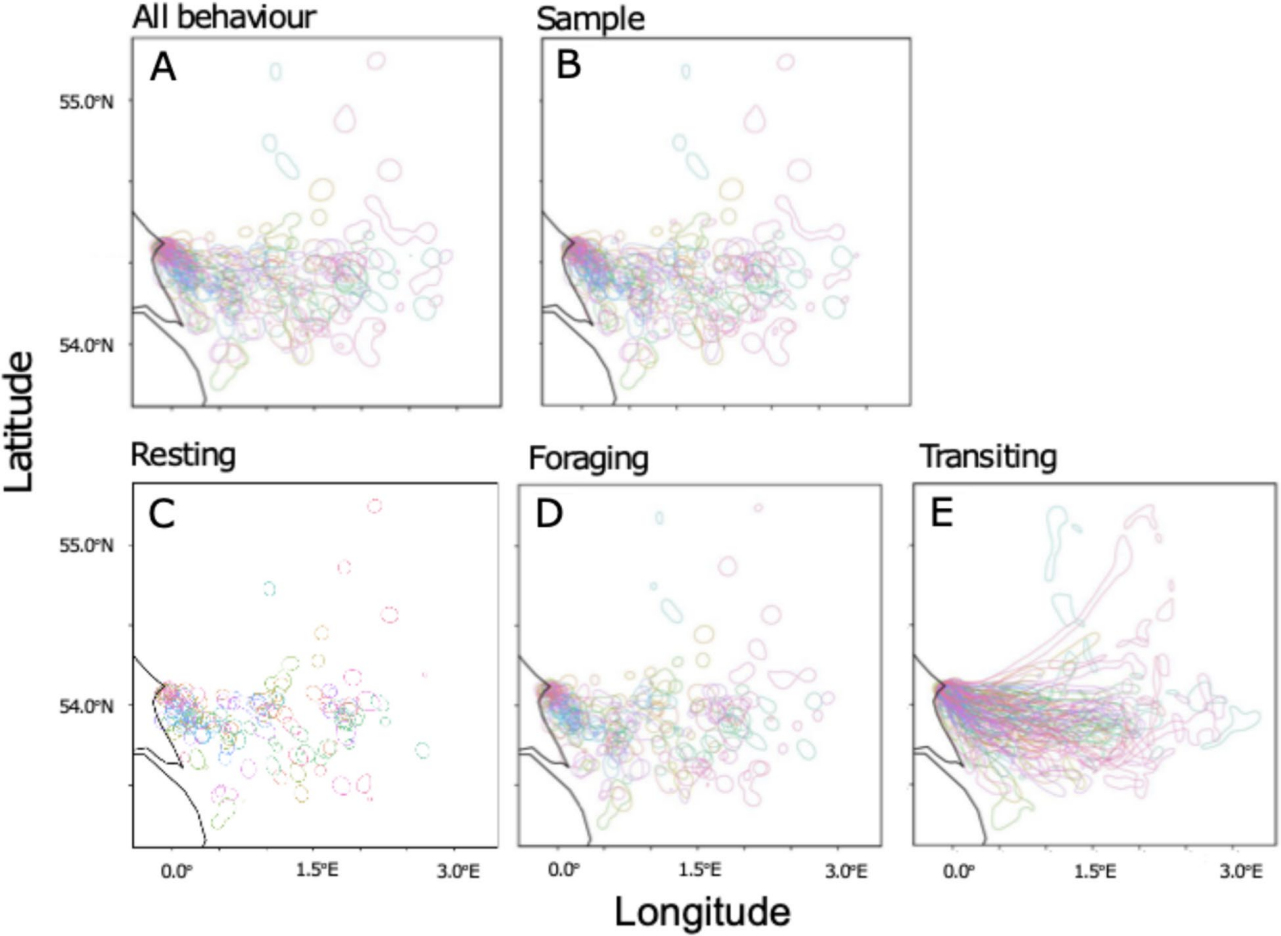
Example of kernel density estimates from kittiwake GPS tracks taken from birds breeding at Bempton Cliffs, England (Fig. 1). Total ‘all behaviour’ tracking data (**A**), Sample: control kernel density estimates based on random subsampling (**B**), and separated behaviours showing resting (**C**), foraging (**D**) and transiting (**E**). The 50% utilisation distribution of each individual is represented in a different colour

We calculated kernel density estimates at the individual-level using all complete trips [108]. Firstly, we calculated utilisation distributions using an individual’s complete ‘all behaviour’ movement data (Fig. 2a). Secondly, we calculated utilisation distributions from an individual’s separated behaviours: resting, foraging, and transiting (Fig. 2b–d). Kernel density estimates require a smoothing parameter (*h*) to determine a probability surface of space use. We used the findScale function in the track2KBA package (v. 1.0.5, [6]) and the log of the median foraging range (*mag,* appropriate for central-place foragers) to provide suitable values reflecting the scale of kittiwake movement bouts (see suppl. info. Appendix 3 Table S1.4 for values of *mag* found for each colony). Additionally, to examine potential changes in the kernel density area due to changes in the number of location fixes within each behaviour, i.e. some behaviours being more frequent than others, we also generated kernel density estimates for each individual using a random subsample of locations across a track. The number of locations in the subsample was equal to the number of locations of the most infrequently recorded behaviour for that individual (Fig. 2e). This control dataset is referred to as the ‘sample’ category from here on.

To quantify whether the kernel density estimates for each individuals’ separate behaviours were captured within an individual’s ‘all behaviour’ kernel density estimate, we calculated the proportion of an individual’s resting, foraging and transiting kernel densities that occurred within their ‘all behaviour’ kernel density. As a control, we also did this using the ‘sample’ kernel densities in place of the separated behavioural kernel densities. To estimate whether the proportion of each behavioural kernel density captured by the total ‘all behaviour’ kernel density differed by behaviour, we used a mixed-effects beta-regression model fitted using the R package glmmTMB (v. 1.1.7, [13]). In this model, we included colony as a random effect and transformed the proportions to avoid strict 0 or 1 values $\dot{x} = \frac{x(N - 1) + 0.5}{N}$ where $N$ is sample size and *x* is the proportion of each behaviour captured within the kernel density of ‘all behaviour’ (Smithson and Verkuilen, 2006).

To explore how the choice of utilisation distribution might affect the representation of different behaviours within ‘all behaviour’ kernel density estimates, we conducted the analysis based on 50% (‘core area’), 75% (‘intermediate area’) and 95% (‘home range’) utilisation distributions. For some colonies the grid size created within estSpaceUse in the Track2KBA package was too small to allow larger kernel density estimates to be mapped (e.g. 95%). In these cases, kernel density surfaces were created using adehabitat with grids increased from a 5% margin to a 20% margin using the function estSpaceUse.

#### Size estimates of population-level key areas

Following the track2KBA workflow we estimated the size of population-level key areas for all behaviour, resting, foraging and transiting and the random sample. To calculate population-level key area estimates we followed BirdLife’s method which combines the kernel density estimates of all tracked individuals at a colony (based on utilisation distributions and calculated above). Using the findSite function in the track2KBA package (v. 1.0.5, [6]) the proportion of individual core areas overlapping in each grid cell were multiplied by the proportional representativeness of the tracking data to give a scaled estimate of the proportion of the source population predictably using the grid cells around each colony. Population, or colony-level, key areas were then delineated by grouping together grid cells used by a threshold percentage of the source population (10%). To estimate the representativeness of the datasets at each colony we used the repAssess function in the track2KBA package (v. 1.0.5, [6], see suppl. info. Appendix 2). Colonies were excluded from further analysis (n = 5) if sample size was too small to calculate representativeness, or representativeness was below the 70% threshold for making population-level estimates [78]. The mean value of representativeness for the remaining colonies was high for ‘all behaviour’ (95.65% ± 4.05), transiting (96.21% ± 4.03), and foraging (92.50% ± 10.23), but lower for resting (84.71% ± 8.77). Details of representativeness for each colony are provided in suppl. info. Appendix 2, Table S2.1.

To compare the spatial extent (km^2^) of colony-specific population-level key areas estimated from ‘all behaviour’ tracking data versus the tracking data split into separated behavioural states (resting, foraging, and transiting), we used a mixed effect model fitted using the R package lme4 (v. 1.1-36, Bates et. al., 2015). In this model, the size of colony-specific key areas transformed by log_10_ was included as the response variable, behaviour was included as a fixed effect, and colony was included as a random effect to account for unexplained variation, e.g. driven by colony size (model structure and outputs detailed in suppl. info. Appendix 5). Diagnostic plots were checked using the R package performance (v. 0.13.0, Lüdecke et al., 2021) to ensure all model assumptions were met. To explore how the choice of utilisation distribution might affect the representation of different behaviours, we repeated the analysis using 50% (‘core area’), 75% (‘intermediate area’) and 95% (‘home range’) utilisation distributions.

## Results

### Tracking data including kittiwake trip metrics

After filtering our tracking data to remove incomplete trips, the dataset comprised 1930 trips from 566 individuals and 14 colonies (Fig. 1). The mean number of trips per individual was 3.41 (Standard Deviation ± 2.33). Mean trip characteristics were: total distance = 100.51 km (SD ± 103.97), maximum distance from colony = 31.90 km (SD ± 30.44), trip duration = 7.11 h (SD ± 7.29), and number of GPS fixes per trip = 226.42 (SD ± 314.5). Colony-specific metrics are provided in suppl. info. Appendix 3, Table S3.1.

### Classifying movement behaviour

**Fig. 3:**
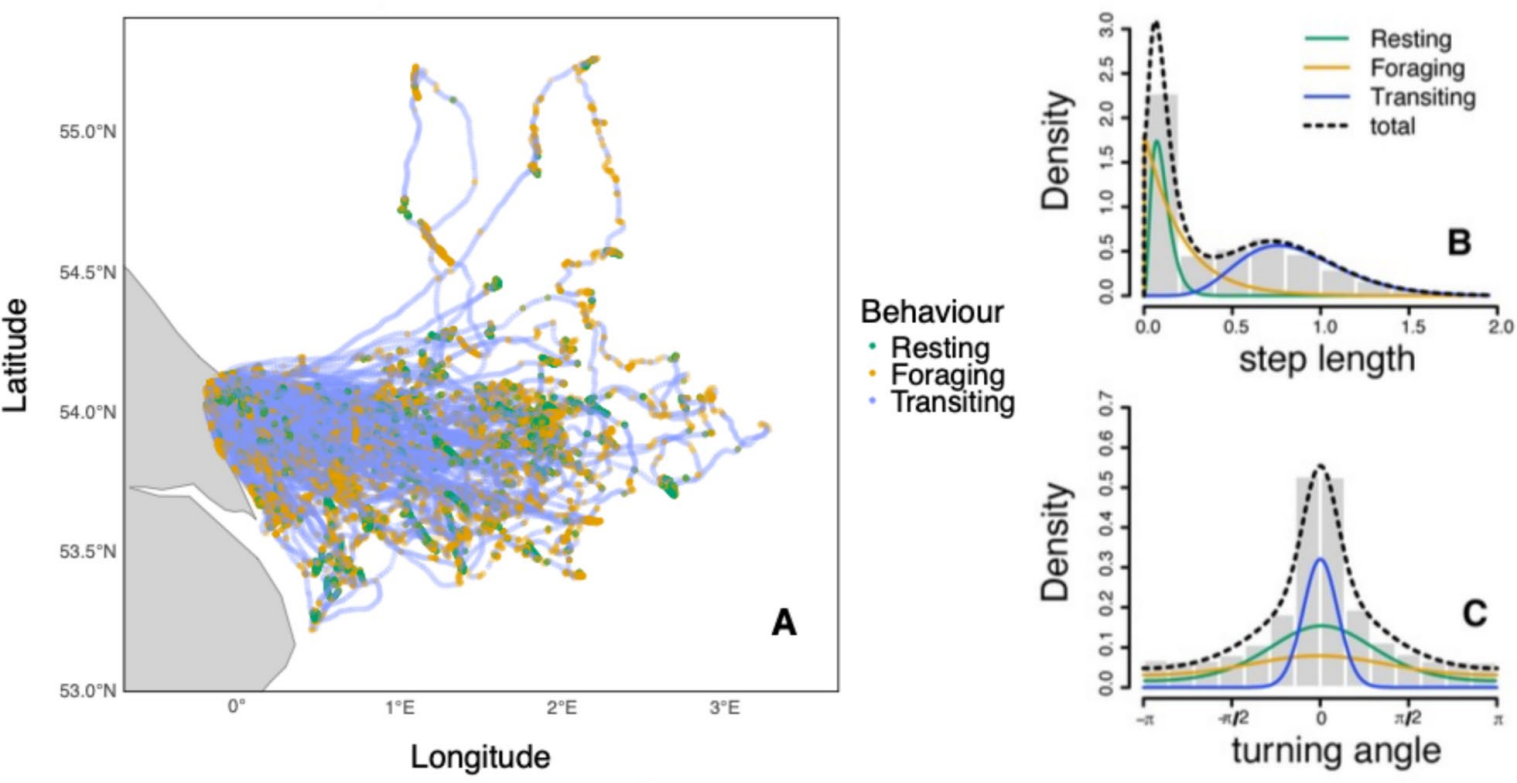
Example of GPS tracks taken from kittiwakes breeding at Bempton Cliffs, England (Fig. 1). The tracks have been separated into resting (green), foraging (orange) and transiting (blue) behaviours (**A**), based on the distribution of step lengths (**B**) and turning angles (**C**)

At-sea behaviour of kittiwakes was classified into resting, foraging and transiting behaviour. Resting behaviour was characterised by short step lengths (mean step length: 0.07 ± 0.03 km, standard deviation in step length: 0.04 ± 0.02) and narrow turning angles (μ = 0 ± 0.01, concentration κ = 10.77 ± 10.69) indicative of low travelling speeds and little directionality. Foraging was categorised as having short to medium step lengths (mean step length: 0.23 ± 0.10 km, standard deviation in step length: 0.26 ± 0.10) and wide turning angles (μ = 0.03 ± 1.19, κ = 0.34 ± 0.20) indicative of medium travelling speeds and low directionality. Finally, transiting was defined by long step lengths (mean step length: 0.98 ± 0.11 km, standard deviation in step length: 0.32 ± 0.05) and narrow turning angles (μ = 0.00 ± 0.01, κ = 9.84 ± 6.59) indicative of fast travelling speeds and strongly directional movement (Fig. 3, suppl. info. Appendix 3, Table S3.2). Step lengths and turning angles for each behaviour showed little variation across colonies (for individual colony breakdown suppl. info. Appendix 3, Table S3.4). Behavioural proportions at sea remained consistent across colonies: kittiwakes spent almost half of their time (weighted grand mean for all colonies: 48, SD ± 6%) foraging, almost a third of their time transiting (29, SD ± 3%), and just less than a quarter of their time resting (23, SD ± 6%). Weighted means were calculated to account for differences in colony size, for details and separate values for each colony see suppl. info. Appendix 3, Table S3.4.

### Comparing key area estimates using ‘all behaviour’ track data versus separated behaviours

#### Kernel density estimates

**Fig. 4:**
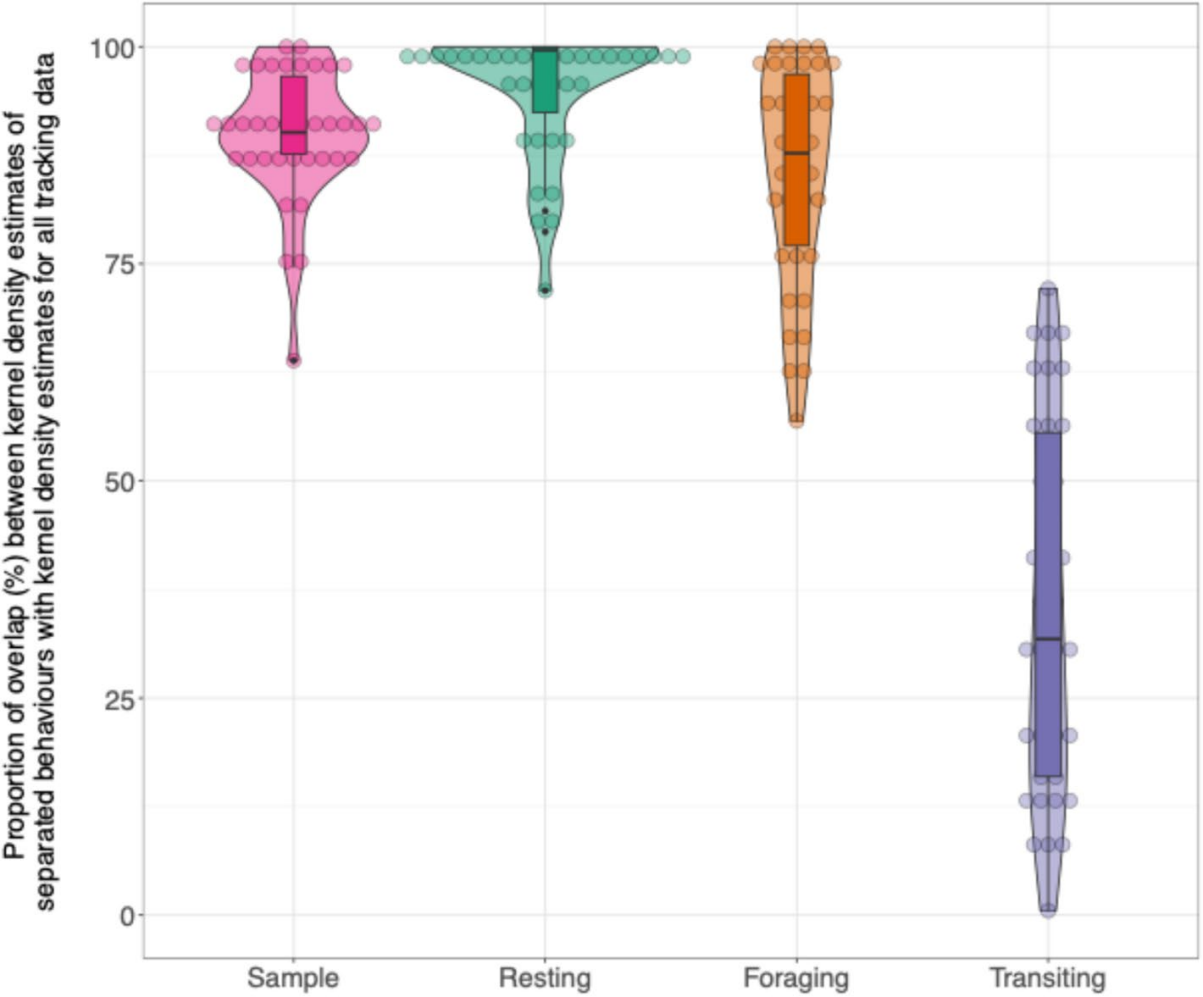
Violin plots of the proportion overlap of the kernel density estimates (based on a 50% utilisation distribution) of each separated behaviour and the random sample of locations, with the kernel density estimates based on all behaviour. Example taken from Coquet, England. Box and whiskers show the overall mean and interquartile range in the proportion of overlap for each behaviour. Each circle within the violins represents an individuals’ kernel density estimate

Kernel density estimates were created for the 50% utilisation distribution of ‘all behaviour’, resting, foraging and transiting and the ‘sample’ category for each individual (Fig. 4). The 50% kernel density estimates derived from ‘all behaviour’ tracking data adequately captured the 50% utilisation distributions of resting (grand mean and standard deviation for all colonies, weighted by number of individuals: 87%, SD ± 10%) and foraging (92%, SD ± 3%) but poorly represented the distribution of transiting behaviour (40%, SD ± 8%). This pattern was consistent across colonies (Appendix 4, Table S4.1). Control kernel density estimates based on the ‘sample’ data were well captured within ‘all behaviour’ kernel density estimates (92%, SD ± 1%), demonstrating that poor capture of behaviours is unlikely to reflect a reduced number of location fixes in separated behaviours. Statistical modelling confirmed that the 50% kernel density estimates derived from ‘all behaviour’ tracking data represented a lower proportion of transiting behaviour (model estimates: 0.4; 95% CI 0.36–0.45, p < 0.001) compared to the ‘sample’ category. The proportion of resting (model estimate: 0.91; 95% CI 0.90–0.93, p < 0.001) and foraging (model estimate: 0.91; 95% CI 0.89–0.92, p < 0.001) were better represented. The control kernel density estimates based on the ‘sample’ category showed an estimated overlap of 0.86 (95% CI 0.84–0.88; p < 0.001). For full model details see suppl. info. Appendix 4, Table S4.3 to S4.4, and Fig. S4.1 and S4.2. We note that the component behaviours of the total ‘all behaviour’ kernel densities are non-independent. For example, if one behaviour was highly clustered, one of the other behaviours would necessarily have to be diffuse to give the observed ‘all behaviour’ distribution, and we discuss this further in the supplementary materials (suppl. info. Appendix 4, Fig. S4.1). We repeated this analysis with tracking data which had been interpolated to 100 s and found minimal difference from non-interpolated results (Appendix 4, Tables S4.6–S4.8).

Kernel density estimates of ‘all behaviour’ created using 75% and 95% utilisation distributions better captured each of the separate behaviours than those using a 50% utilisation distribution. Transiting remained less well captured compared to resting, foraging and the ‘sample’ category, however the overall proportion of transiting captured within the ‘all behaviour’ kernel density estimate improved with increasing utilisation distribution (50% utilisation distribution (UD) = 0.4, 75% UD = 0.61, 95% UD = 0.86). For kernel density estimates based on a 75% utilisation distribution, transiting behaviour was the least well captured behavioural state (model estimates: 0.61; 95% CI 0.58–0.64), compared to resting (model estimate: 0.96; 95% CI 0.95–0.96) and foraging (model estimate: 0.95; 95% CI 0.95–0.96) and the ‘sample’ category (model estimate: 0.90; 95% CI 0.88–0.91). For kernel density estimates based on a 95% utilisation distribution, transiting behaviour was still the least well captured behavioural state (model estimates: 0.86; 95% CI 0.85–0.88) compared to resting (model estimate: 0.97; 95% CI 0.97–0.98) and foraging (model estimate: 0.97; 95% CI 0.96–0.97) and the ‘sample’ of all behaviour (model estimate: 0.95; 95% CI 0.94–0.95). For full model outputs see suppl. info. Appendix 4, Tables S4.5 to S4.11, and Fig. S4.3 and S4.4.

#### Size estimates of population-level key areas

**Fig. 5:**
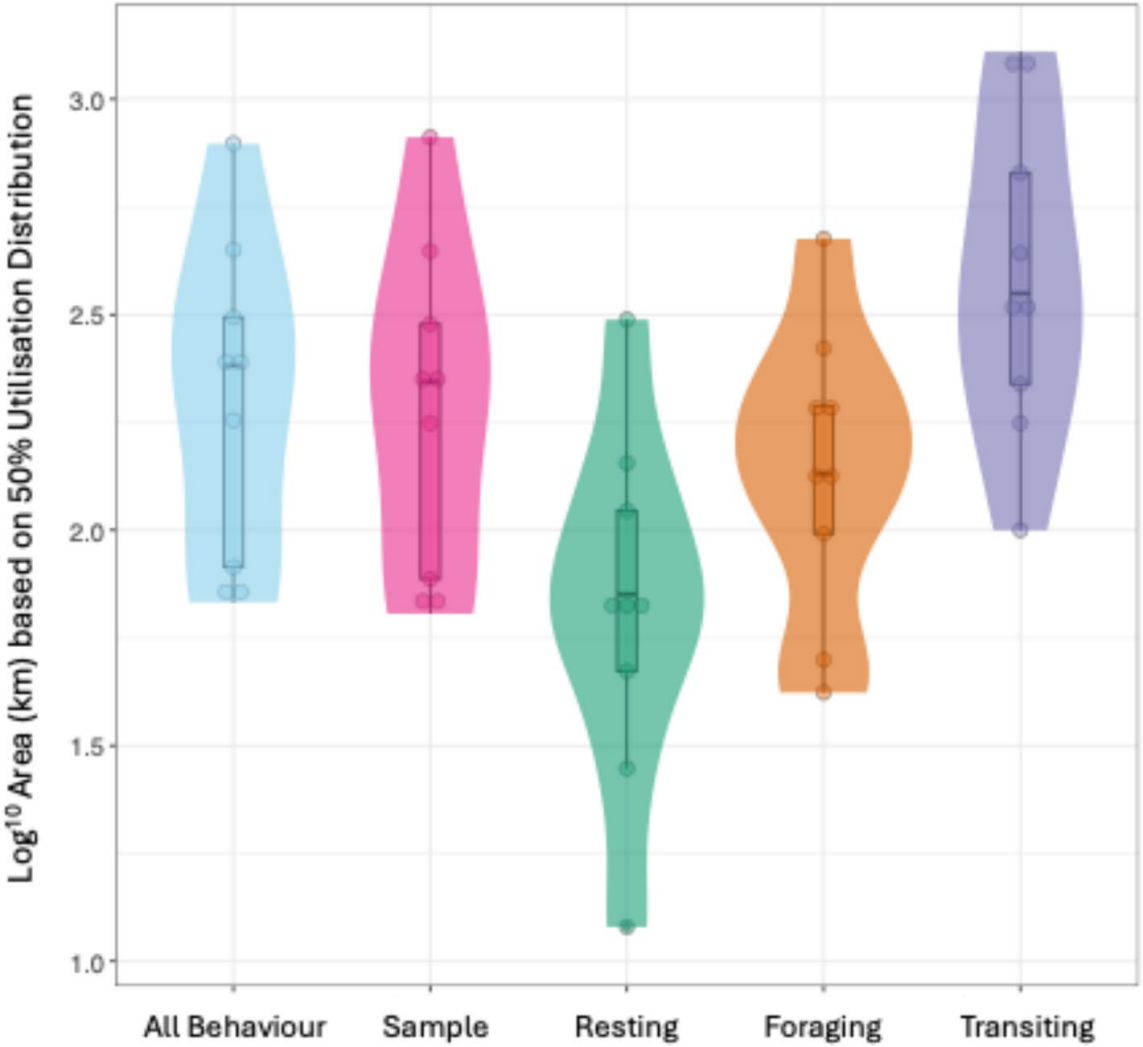
Violin plots of the spatial extent of population-level key area estimates (based on a 50% utilisation distribution) for ‘all behaviour’, a random sample of ‘all behaviour’, and tracking data separated into resting, foraging, and transiting behavioural states. Each circle within the violins represents a different colony, box and whiskers show the overall mean and interquartile range

The estimated size of population-level key areas was larger for transiting behaviour (mean average across colonies: 681 km^2^) than for ‘all behaviour’ (380.11 km^2^), resting (133.89 km^2^) and foraging (249.56km^2^, individual colony values in suppl. info. Appendix 5, Table S5.1). The results of our mixed effect model indicated that population-level key areas for transiting were significantly different, and nearly 30% larger than those estimated using ‘all behaviour’ data (0.28; 95% CI 0.12, 0.45 p = 0.001). By contrast, key areas for resting (− 0.48; 95% CI − 0.65, − 0.31; p < 0.001) behaviour were smaller than those derived from ‘all behaviour’ data (Fig. 5). The extent of the key area for kernel density estimates of foraging (− 0.16; 95% CI − 0.33, − 0.00; p > 0.05) and the ‘sample’ of locations did not differ from key areas based on ‘all behaviour’ data (− 0.02; 95% CI − 0.19, − 0.15; p > 0.05, Marginal R^2^ 0.314, Conditional R^2^ 0.846). Full model results are detailed in suppl. info. Appendix 5, Table S5.2, Table S5.3 and Fig. S5.1 and S5.2.

In population-level estimates of key areas estimated using 75% utilisation distributions, key transiting areas were still larger than for ‘all behaviour’ (0.18; 95% CI 0.07–0.29; p < 0.01), with areas of resting (− 0.36; 95% CI − 0.47–− 0.25; p < 0.001), and foraging (− 0.20 95% CI − 0.32–− 0.09; p < 0.01), smaller that for ‘all behaviour’, and no difference in the sample (− 0.04; 95% CI − 0.15–− 0.07; p > 0.01). In estimates based on 95% utilisation distributions, population-level key areas for transiting were not significantly different from key areas based on ‘all behaviour’ (0.03; 95% CI 0.12–0.50; p > 0.01). Key areas of resting (− 0.37; 95% CI − 0.49–− 0.25; p < 0.001) and foraging (− 0.25; 95% CI − 037–− 0.13; p < 0.001) were smaller than ‘all behaviour’ and there was no difference in the size of the ‘sample’ key area (− 0.04; 95% CI 0.16–0.08; p > 0.01). Full model results are detailed in suppl. info. Appendix 5, Tables S5.4 to S5.9 and Figs. S5.3 and S5.4.

## Discussion

Effective identification of key areas that are critical for species persistence is essential for protected area designation, mitigation of anthropogenic threats, and sustainable land- and sea-use management [18, 45, 62, 77, 82]. A common method for delineating these conservation areas is to identify regions where animals occur in high-density [4, 6, 78, 108]. This approach does not account for variation in threat exposure associated with different behavioural states, and risks underestimating threats that occur during periods of lower aggregation. The present study demonstrates that individual-based kernel density estimates derived from composite “all behaviour” movement data effectively capture resting and foraging behaviours in part by delineating an area larger than the areas required for each of these behaviours. In contrast, the composite “all behaviour” data fails to accurately represent around half of the area required for transiting behaviours and the area required for transiting alone tends to be larger than the composite “all behaviour” area. These findings suggest that incorporating behavioural-state analysis into key area assessments can enhance conservation planning by more precisely identifying locations where individuals are more susceptible to different kinds of threats.

The results presented here indicate that standard methodologies for identifying key areas, which do not differentiate behavioural modes, may underestimate the extent to which kittiwakes are exposed to threats whilst transiting. While key areas for foraging and resting were well captured within individual-level “all behaviour” kernel density estimates, those for transiting behaviour were spatially distinct. Additionally, when estimates were scaled up to the population-level, key areas of transiting behaviour were larger than those for other behaviours, as well as for all behaviours combined. Kittiwakes have been found to be particularly vulnerable to collisions with offshore wind farms due to their flight heights and relatively high proportion of time spent flying [52, 54]. The spatial distribution of transiting behaviour along ‘commuting corridors’ between breeding and foraging locations may therefore necessitate separate consideration during marine spatial planning, especially given the projected expansion of offshore renewable energy infrastructure, particularly in the United Kingdom (from 8 GW in 2020 to a targeted 40 GW by 2030 [8,65]).

For kittiwakes, key population-level areas for resting and foraging were smaller than those identified from the “all behaviour” tracking dataset (across all utilisation distributions), indicating potential to refine targeted areas for behaviour-specific threat mitigation. In some species, these densely distributed behaviours can increase exposure to specific threats. For example, foraging northern gannets (*Morus bassanus*) face an elevated risk of collision when plunge-diving near wind turbines, whilst benthic foraging and deep-diving species (e.g. auks) are particularly vulnerable to tidal and wave energy devices [24, 51]. The results of this study indicate that both individual and population-level key area estimates based on larger utilisation distributions (i.e. 75% and 95%, rather than 50%), better capture the distribution of transiting behaviour. For species which are particularly vulnerable when transiting (e.g. kittiwakes) or where transiting is the dominant behaviour (e.g. species which forage on the wing or disperse widely), larger utilisation distributions may be more suitable for capturing key areas and estimating threat exposure. While adopting a broader spatial scale for protection may better encapsulate transiting behaviour, it necessitates a trade-off between scale and resolution of conservation effort. As allocation of conservation resources is constrained by financial and logistical limitations, prioritising the most ecologically significant habitats is essential to ensuring effective management [2, 93]. Conservation interventions that fail to align with ecological needs risk misallocation of resources, stakeholder resistance, and suboptimal conservation outcomes [48, 56]. Behavioural-state analysis, selection of utilisation distributions at an appropriate scale, and an understanding of species-specific responses to threats can therefore support marine spatial planning through the design of more targeted conservation zones with mitigation strategies that prioritise essential behaviours while minimising spatial conflict with other marine industries [1, 39, 57, 97, 100].

Separating movement data by behaviour supports the delineation of targeted areas for conservation, however the distribution of specific behaviours may change over time as a result of intrinsic and extrinsic factors. This variation may be caused by seasonal, diurnal or circadian changes [15, 70, 98], underlying individual variation in decision making and habitat preferences [91, 111], or in response to environmental change [21, 46, 71]. Additionally, distribution may change with population structure, as behaviours can be exhibited unequally by different sex or age classes [3, 7, 75]. To ensure that spatial and temporal limits for conservation efforts are future proof, conservation practitioners must account for these factors when planning protective actions. An understanding of species-specific site and route fidelity can support marine spatial planning decisions and threat assessment through the choice of appropriate utilisation distributions (e.g. larger where spatial distribution is more variable or transiting is dominant). Dynamic conservation measures that incorporate real-time environmental data and behaviour-specific distribution models could allow spatial planners and policy makers to respond accordingly, adapting protection to track changes in the distribution of key areas [31, 53, 104].

Incorporating behavioural-state into estimates of space use can help guide not just the spatial placement of static (place-based) protected areas, but also the timing and location of seasonal or spatially dynamic protection. By recognising variation in space-use by behaviour, conservation practices can be tailored to reflect changes in threat exposure, minimise negative ecological consequences and stakeholder conflict, and maximise conservation resources [3, 7, 87, 97]. For example, implementing spatial restrictions on industrial fishing within seabird foraging zones during the breeding season when individuals are constrained by central place foraging [64] or wind turbine curtailment during peak migratory periods or if adverse environmental conditions increase spatial overlap with key foraging areas.

## Conclusion

Our study highlights the importance of incorporating behavioural-state differentiation when identifying key spatial areas for wide-ranging species such as seabirds. Standard approaches that rely on aggregated movement data may fail to capture transiting behaviour, thereby underestimating exposure to anthropogenic threats. Using movement data from black-legged kittiwakes, we demonstrate that key areas derived from total movement datasets do not fully represent the distribution of different at-sea behaviours. While adopting a broader spatial scale for protection may better encapsulate transiting behaviour, it necessitates a trade-off between scale and resolution of conservation effort. As individuals may be more susceptible to specific threats while exhibiting certain behaviours, incorporating behavioural data into the designation of protected areas and environmental impact assessments could help minimise negative interactions between seabirds and human activities, focusing conservation resources while minimising stakeholder spatial conflict with marine industries. For instance, the planning of offshore wind farm developments should prioritise avoidance of key transiting areas to reduce mortality risks associated with turbine collisions. Our results have immediate applications for spatial planning and the designation and management of effective protected areas, particularly in the context of mitigating siting and operational risks associated with offshore wind energy developments. More broadly, the findings highlight the need for a nuanced understanding of species-specific behaviour to refine conservation strategies and improve ecological impact assessments. A better understanding of spatiotemporal overlap between seabird behaviours and environmental stressors could improve the effectiveness of regulatory frameworks governing marine spatial planning.

## Supplementary Information

Additional file 1.

## Data Availability

The dataset supporting the conclusions of this article is part of an open dataset made available by RSPB under a Creative Common Attribution (CC BY 4.0) license. The tracking data used in the current manuscript can be viewed at the Seabird Tracking Database (BirdLife International 2023) ([http://seabirdtracking.org/] (http://seabirdtracking.org) mapper/contributor.php? contributor\_id = 950. Data Owner/contact: RSPB Data Unit. Tracking data can be obtained upon request by contacting the RSPB Data Unit. Code for analysis is available in the repository KDEs at [https://github.com/hannahwood25/KDEs.git] (https://github.com/hannahwood25/KDEs.git). It was written in the programming language R using the software R (version 3.2.2, R Core Team, 2021) in R Studio (version 2023.06.0 + 421).

## Abbreviations

FAME: Future of the Atlantic Marine Environment
GPS: Global Positioning System
HMMs: Hidden Markov models
KBAs: Key Biodiversity areas
KDEs: Kernel Density Estimates
MPAs: Marine Protected Areas
RSPB: Royal Society for the Protection of Birds
STAR: Seabird Tracking and Research
SD: Standard Deviation
