# Supplementary material for: A behavioural approach to key area identification in seabirds for threat mitigation and spatial management
Source: Anim Biotelemetry. 2025 Oct 21;13(1):34. doi: 10.1186/s40317-025-00427-z (PMC12576929; doi:10.1186/s40317-025-00427-z)
Supplement: Supplementary file 1 — Additional file 1. [file 40317_2025_427_MOESM1_ESM.docx]

# Supplementary Information: A Behavioural Approach to Key Area Identification in Seabirds for Threat Mitigation and Spatial Management

Table of Contents

[Supplementary Information: A Behavioural Approach to Key Area Identification in Seabirds for Threat Mitigation and Spatial Management 1](#_Toc206071712)

[Appendix 1: Methods 7](#_Toc206071713)

[Table S1.1 Summary of original FAME/STAR tagging data before filtering. 7](#_Toc206071714)

[Table S1.2 Tagging years in colonies with over 8 tagged individuals. Some individuals were removed after trip segmentation due to incomplete trips. 8](#_Toc206071715)

[Table S1.3 Starting parameters for behavioural classification based on step lengths and turning angles used in hidden Markov models. 8](#_Toc206071716)

[Figure S1.1 Flowchart illustrating the pipeline of analysis for this study. 9](#_Toc206071717)

[Table S1.4 Smoothing parameters for the kernel density estimates (values of mag) for each colony) 10](#_Toc206071718)

[Appendix 2. Representative assessment values 11](#_Toc206071719)

[Table S2.1 Representativeness values for behaviours at each colony indicating the degree to which the tracking data represents the distribution of the source population. The proportion (inclusion rate) approximates the specified utilization distribution quantile when the tracked sample is fully representative (e.g., approximately 50% of locations for the entire source population would be expected to fall within the 50% utilisation distribution of the sample). According to Lascelles et al., (2016) values over 70% are representative enough to make population-level estimates. 12](#_Toc206071720)

[Table S2.2 Mean average and standard deviation of representativeness values for colonies included in estimates of population-level key areas 12](#_Toc206071721)

[Appendix 3. Results: Trip metrics and behaviour 13](#_Toc206071722)

[Table S3.1 Summary of kittiwake tracking data and trip metrics at each colony after removing incomplete trips and small colonies. 13](#_Toc206071723)

[Table S3.2 Average Step lengths and turning angles of different kittiwake behavioural states at all colonies. 13](#_Toc206071724)

[Table S3.3 Mean average and standard deviation (SD) of step lengths and mean turning angle and concentration of different kittiwake behaviours classified using a hidden Markov model for each colony. 14](#_Toc206071725)

[Table S3.4 Proportion of time spent in each behaviour at each colony. 15](#_Toc206071726)

[Appendix 4. Kernel Density Estimate Results 16](#_Toc206071727)

[Table S4.1 Percentage of kernel density estimates of separated behaviours captured by “all behaviour” kernel density estimates. 16](#_Toc206071728)

[Table S4.2 Range and weighted grand mean and standard deviations of proportions of separate behaviour kernel density estimates captured within all behaviour kernel density. 17](#_Toc206071729)

[Figure S4.1 Model structure for predicting proportional overlap of the ‘all behaviours’ kernel with the behaviours within it. 17](#_Toc206071730)

[Table S4.3 Predicted proportions of each behavioural KDE captured by All Behaviour KDE using utilisation distributions of 50%. 17](#_Toc206071731)

[Table S4.4 Model outputs from mixed effects beta regression comparing the proportion of each behavioural kernel density captured by the total ‘all behaviour’ kernel density using utilisation distributions of 50%. The model intercept shows the estimated proportional overlap in area between ‘sample’ and ‘all behaviour’. 18](#_Toc206071732)

[Table S4.5 Random effects from mixed effects beta regression comparing the proportion of each behavioural kernel density captured by the total ‘all behaviour’ kernel density using utilisation distributions of 50%. 18](#_Toc206071733)

[Following Trevail et al (2019) tracking data in this study were not interpolated. According to MoveHMM package guidance: ‘Small violations of the assumption of regular time intervals and negligible measurement error can be accommodated by moveHMM, e.g., GPS measurement error may be treated as negligible if it is small compared to the scale of movement steps.’ All tracking data were all collected on igotU devices programmed to record every 100 seconds. Overall, mean average time interval for the dataset was 104.3 seconds (standard deviation 9.33). 18](#_Toc206071734)

[Table S4.6 Predicted proportions of each behavioural KDE captured by All Behaviour KDE using utilisation distributions of 50% after tracking data was interpolated. 18](#_Toc206071735)

[Table S4.7 Model outputs from mixed effects beta regression comparing the proportion of each behavioural kernel density captured by the total ‘all behaviour’ kernel density using utilisation distributions of 50%. The model intercept shows the estimated proportional overlap in area between ‘sample’ and ‘all behaviour’ after tracking data was interpolated. 19](#_Toc206071736)

[Table S4.8 Random effects from mixed effects beta regression comparing the proportion of each behavioural kernel density captured by the total ‘all behaviour’ kernel density using utilisation distributions of 50% after tracking data was interpolated. 19](#_Toc206071737)

[Table S4.9 Predicted proportions of each behavioural KDE captured by All Behaviour KDE using utilisation distributions of 75%. 19](#_Toc206071738)

[Table S4.10 Model outputs from mixed effects beta regression comparing the proportion of each behavioural kernel density captured by the total ‘all behaviour’ kernel density using utilisation distributions of 75%. The model intercept shows the estimated proportional overlap in area between ‘sample’ and ‘all behaviour’. 20](#_Toc206071739)

[Table S4.11 Random effects from mixed effects beta regression comparing the proportion of each behavioural kernel density captured by the total ‘all behaviour’ kernel density using utilisation distributions of 75%. 20](#_Toc206071740)

[Table S4.12 Predicted proportions of each behavioural KDE captured by All Behaviour KDE using utilisation distributions of 95%. 20](#_Toc206071741)

[Table S4.13 Model outputs from mixed effects beta regression comparing the proportion of each behavioural kernel density captured by the total ‘all behaviour’ kernel density using utilisation distributions of 95%. The model intercept shows the estimated proportional overlap in area between ‘sample’ and ‘all behaviour’. 20](#_Toc206071742)

[Table S4.14 Random effects from mixed effects beta regression comparing the proportion of each behavioural kernel density captured by the total ‘all behaviour’ kernel density using utilisation distributions of 95%. 21](#_Toc206071743)

[Figure 4.2 Model outputs of proportion of behavioural KDEs captured by All Behaviour KDE using a 50% utilisation distribution. A: Estimated values for proportion of overlap, B: predicted values, C: Random effects. Behaviour 1 and the Intercept are the sub-sample from ‘all behaviour’ 22](#_Toc206071744)

[Figure 4.3 Model outputs of proportion of behavioural KDEs captured by All Behaviour KDE using a 75% utilisation distribution. A: Estimated values for proportion of overlap, B: predicted values, C: Random effects. Behaviour 1 and the Intercept are the sub-sample from ‘all behaviour’ 22](#_Toc206071745)

[Figure 4.4 Model outputs of proportion of behavioural KDEs captured by All Behaviour KDE using a 95% utilisation distribution. A: Estimated values for proportion of overlap, B: predicted values, C: Random effects. Behaviour 1 and the Intercept are the sub-sample from ‘all behaviour’ 23](#_Toc206071746)

[Appendix 5: Size Estimates of Population-level Key Areas 24](#_Toc206071747)

[Table S5.1 Size of key areas, defined using 50% utilisation distribution, of all behaviour, three separated behaviours (resting, foraging, and transiting) and a sample of all behaviours as small as the smallest behaviour. 24](#_Toc206071748)

[Figure S5.1 Model structure for linear mixed effect model to compare the spatial extent (in km^2^) of key area estimates based on a 50% utilisation distribution and calculated using ‘all behaviour’ track data, separated behaviour track data and a control ‘sample’. We included ‘colony’ as a random effect and area estimates were logged. 24](#_Toc206071749)

[Table S5.2 Model outputs from linear mixed effects model comparing the spatial extent of delineated key areas 50% utilisation distribution. Site: Sample is a random subsample of all behaviour. 24](#_Toc206071750)

[Table S5.3 Random effects from linear mixed effects model comparing the spatial extent of delineated key areas based on a 50% utilisation distribution. 25](#_Toc206071751)

[Figure 5.2 Predicted spatial extent of population-level key areas based on a 50% utilisation distribution from linear mixed effects model. All Behaviour includes all tracking data, ‘Sample’ is a random subsample of the tracking data, Foraging, Resting and Transiting behaviours were defined from tracking data using hidden Markov models. Natural log was removed for the predictive values. 25](#_Toc206071752)

[Table S5.4 Size of key areas defined using 75% kernel density estimates of all behaviour, three separated behaviours (resting, foraging, and transiting) and a sample of all behaviours as small as the smallest behaviour. 26](#_Toc206071753)

[Table S5.5 Model outputs from linear mixed effects model comparing the spatial extent of delineated key areas based on 75% utilisation distribution. Site: Sample is a random subsample of all behaviour. 26](#_Toc206071754)

[Table S5.6 Random effects from linear mixed effects model comparing the spatial extent of delineated key areas based on a 75% utilisation distribution. 26](#_Toc206071755)

[Figure 5.3 Violin plots of the spatial extent of population-level key area estimates, based on a 75% utilisation, for ‘all behaviour’, a random sample of ‘all behaviour’, and tracking data separated into resting, foraging, and transiting behavioural states. Each circle within the violins represents a different colony, box and whiskers show the overall mean and interquartile range. 27](#_Toc206071756)

[Table S5.7 Size of key areas defined using 95% kernel density estimates of all behaviour, three separated behaviours (resting, foraging, and transiting) and a sample of all behaviours as small as the smallest behaviour. 27](#_Toc206071757)

[Table S5.8 Model outputs from linear mixed effects model comparing the spatial extent of delineated key areas 95% utilisation distribution. Site: Sample is a random subsample of all behaviour. 28](#_Toc206071758)

[Table S5.9 Random effects from linear mixed effects model comparing the spatial extent of delineated key areas based on a 95% utilisation distribution. 28](#_Toc206071759)

[Figure 5.4 Violin plots of the spatial extent of population-level key area estimates, based on a 95% utilisation, for ‘all behaviour’, a random sample of ‘all behaviour’, and tracking data separated into resting, foraging, and transiting behavioural states. Each circle within the violins represents a different colony, box and whiskers show the overall mean and interquartile range. 29](#_Toc206071760)

# Appendix 1: Methods

## Table S1.1 Summary of original FAME/STAR tagging data before filtering.

| **Colony** | **Latitude** | **Longitude** | **No. of individuals** | **No. of years** |
| --- | --- | --- | --- | --- |
| Bardsey | 52.75831 | -4.78088 | 8 | 1 |
| Bempton | 54.11397 | -0.07802 | 98 | 6 |
| Bullers of Buchan | 57.43236 | -1.81506 | 5 | 1 |
| Cape Wrath | 58.60205 | -4.76917 | 5 | 1 |
| Coquet | 55.33447 | -1.53724 | 36 | 2 |
| Colonsay | 56.08788 | -6.23985 | 81 | 5 |
| Fair Isle | 59.54438 | -1.62878 | 8 | 4 |
| Filey | 54.21848 | -0.27203 | 47 | 3 |
| Fowlsheugh | 56.92553 | -2.19754 | 15 | 1 |
| Isle of May | 56.18184 | -2.55138 | 49 | 3 |
| Lambay | 53.49505 | -5.99866 | 14 | 2 |
| Orkney: Copinsay | 58.90037 | -2.66629 | 30 | 4 |
| Orkney: Muckle Skerry | 58.68886 | -2.91907 | 47 | 5 |
| Puffin Island | 53.32089 | -4.02554 | 82 | 5 |
| Rathlin | 55.30083 | -6.26964 | 9 | 2 |
| St Abbs | 55.91614 | -2.14000 | 15 | 1 |
| Scilly: St Agnes | 49.89508 | -6.33943 | 4 | 2 |
| Scilly: St Martins | 49.96607 | -6.26362 | 35 | 3 |
| Sula Skerry | 59.08442 | -4.40659 | 4 | 1 |
| Whinnyfold | 57.38383 | -1.87076 | 20 | 1 |

NB Tagging data was filtered to remove colonies with fewer than 8 tagged individuals plus Fair Isle as it has fewer than 3 individuals tagged per year.

## Table S1.2 Tagging years in colonies with over 8 tagged individuals. Some individuals were removed after trip segmentation due to incomplete trips.

| **Colony** | **2010** | **2011** | **2012** | **2013** | **2014** | **2015** | **Total over all years** | **No. of Years** |
| --- | --- | --- | --- | --- | --- | --- | --- | --- |
| Bardsey | 8 |  |  |  |  |  | 8 | 1 |
| Bempton | 22 | 17 | 8 | 19 | 17 | 15 | 98 | 6 |
| Coquet |  | 13 | 23 |  |  |  | 36 | 2 |
| Colonsay | 8 | 26 | 24 | 11 | 12 |  | 81 | 5 |
| Filey |  |  |  | 17 | 16 | 14 | 47 | 3 |
| Fowlsheugh |  |  | 15 |  |  |  | 15 | 1 |
| Isle of May |  |  | 17 | 21 | 11 |  | 49 | 3 |
| Lambay | 10 | 4 |  |  |  |  | 14 | 2 |
| Orkney: Copinsay | 11 | 7 | 8 | 4 |  |  | 30 | 4 |
| Orkney: Muckle Skerry | 8 | 8 | 12 | 7 | 12 |  | 47 | 5 |
| Puffin Island | 15 | 29 | 25 | 4 |  | 9 | 82 | 5 |
| St Abbs |  |  | 15 |  |  |  | 15 | 1 |
| Scilly: St Martins | 18 | 14 | 3 |  |  |  | 35 | 3 |
| Whinnyfold |  |  | 20 |  |  |  | 20 | 1 |
| Total tagged individuals | 100 | 118 | 170 | 83 | 68 | 38 | 577 |  |

## Table S1.3 Starting parameters for behavioural classification based on step lengths and turning angles used in hidden Markov models.

| **Parameter** | **Values** | | | |
| --- | --- | --- | --- | --- |
| **Step length mean** | 0.08 | 0.27 | | 1 |
| **Step length variance** | 0.05 | 0.31 | | 0.35 |
| **Step length parameter** | Step mean 0 | | Step sd 0 | |
| **Turning angle mean** | 0.00 | 0.00 | | 0.00 |
| **Turning angle concentration (Kappa)** | 14 | 0.4 | | 6.8 |
| **Turning angle parameter** | Angle mean 0 | | Kappa 0 | |

## Figure S1.1 Flowchart illustrating the pipeline of analysis for this study.

## Table S1.4 Smoothing parameters for the kernel density estimates (values of mag) for each colony)

| **Colony** | **Median maximum distance** | **Step length** | ***mag*** | **href** |
| --- | --- | --- | --- | --- |
| Bardsey | 18.49 | 0.41 | 2.92 | 2.62 |
| Bempton | 30.74 | 0.19 | 3.43 | 6.61 |
| Coquet | 7.36 | 0.15 | 2 | 2.94 |
| Colonsay | 32.44 | 0.07 | 3.48 | 3.35 |
| Filey | 40.84 | 0.37 | 3.71 | 9.27 |
| Fowlsheugh | 70.03 | 0.24 | 4.25 | 7.74 |
| Isle of May | 12.99 | 0.13 | 2.56 | 3.4 |
| Lambay | 22.09 | 0.23 | 3.1 | 2.37 |
| Orkney: Copinsay | 5.46 | 0.12 | 1.7 | 3.34 |
| Orkney: Muckle Skerry | 3.75 | 0.22 | 1.32 | 1.6 |
| Puffin Island | 9.62 | 0.2 | 2.26 | 2.36 |
| St Abbs | 11.04 | 0.07 | 2.4 | 6.46 |
| Scilly: St Martins | 9.79 | 0.15 | 2.28 | 2 |
| Whinnyfold | 39.19 | 0.14 | 3.67 | 4.07 |

# Appendix 2. Representative assessment values

Estimates of site maps using findSite require estimates of representativity (the degree to which the sample of tracked individuals represented the space use of the wider population at each colony). We calculated this for each colony/behaviour/sample at each colony using the repAssess function in the R package Track2KBA (v. 1.0.5, Beal et al., 2021). We used this approach to calculate representativeness of the tracks in all behaviour as well as each separated behaviour. The repAssess function requires a minimum of four independent tracks, and the estimation of the asymptote estimation is unreliable for small sample sizes (e.g., *n* < 10 independent tracks). Values could not be calculated for some individual behavioural states at four colonies because the number of track locations was too few. These were Bardsey (all separated behaviours), St Abbs (foraging), Fowlsheugh and Lambay (transiting). These samples were therefore excluded from further analysis. The mean average value of representativeness was 91.7% +/- 9.62 for ‘all behaviour’ data; 78.9% +/-15.31 for resting; 83.38% +/- 24.02 for foraging; and 95.28% +/- 4.24 transiting. Sample representativeness at individual colonies in 4.1.

## Table S2.1 Representativeness values for behaviours at each colony indicating the degree to which the tracking data represents the distribution of the source population. The proportion (inclusion rate) approximates the specified utilization distribution quantile when the tracked sample is fully representative (e.g., approximately 50% of locations for the entire source population would be expected to fall within the 50% utilisation distribution of the sample). According to Lascelles et al., (2016) values over 70% are representative enough to make population-level estimates.

| **Colony** | **All behaviour** | **Resting** | **Foraging** | **Transiting** |
| --- | --- | --- | --- | --- |
| Bardsey* | 95.95 | N/A | N/A | N/A |
| Bempton | 96.53 | 88.15 | 95.18 | 97.69 |
| Coquet | 98.86 | 79.83 | 91.60 | 96.70 |
| Colonsay | 99.36 | 89.93 | 99.79 | 96.55 |
| Filey* | 86.27 | 34.81 | 78.60 | 94.65 |
| Fowlsheugh* | 64.38 | 85.66 | 10.60 | N/A |
| Isle of May | 93.75 | 75.22 | 93.11 | 99.54 |
| Lambay* | 98.35 | 75.26 | 78.82 | N/A |
| Orkney: Copinsay | 88.90 | 81.66 | 97.34 | 86.98 |
| Orkney: Muckle Skerry | 98.16 | 95.63 | 94.52 | 99.88 |
| Puffin Island | 98.36 | 96.47 | 97.28 | 99.02 |
| St Abbs* | 78.08 | 67.60 | N/A | 87.54 |
| Scilly: St Martins | 97.55 | 84.76 | 97.62 | 96.43 |
| Whinnyfold | 89.35 | 70.75 | 66.07 | 93.08 |
|  |  |  |  |  |
| **Mean** | **91.70** | **78.90** | **83.38** | **95.28** |
| **Standard Deviation** | **9.62** | **15.31** | **24.02** | **4.24** |

*Sample size too small to calculate representativeness. Removed from further analysis.

## Table S2.2 Mean average and standard deviation of representativeness values for colonies included in estimates of population-level key areas

|  | **All behaviour** | **Resting** | **Foraging** | **Transiting** |
| --- | --- | --- | --- | --- |
| **Mean** | 95.65 | 84.71 | 92.50 | 96.21 |
| **Standard Deviation** | 4.05 | 8.77 | 10.23 | 4.03 |

# Appendix 3. Results: Trip metrics and behaviour

## Table S3.1 Summary of kittiwake tracking data and trip metrics at each colony after removing incomplete trips and small colonies.

| Colony | No. of individuals | No. of trips | Mean total distance | Sd total distance | Mean max distance | Sd max distance | Mean duration | Sd duration |
| --- | --- | --- | --- | --- | --- | --- | --- | --- |
| Bardsey | 8 | 22 | 75.19 | 43.73 | 20.53 | 9.63 | 4.73 | 3.03 |
| Bempton | 95 | 266 | 142.65 | 160.72 | 49.42 | 50.37 | 8.88 | 10.83 |
| Coquet | 36 | 101 | 68.34 | 90.76 | 21.10 | 27.71 | 4.69 | 5.46 |
| Colonsay | 79 | 181 | 146.43 | 148.04 | 33.81 | 26.62 | 15.37 | 15.90 |
| Filey | 44 | 131 | 179.16 | 187.59 | 62.94 | 60.00 | 9.74 | 10.57 |
| Fowlsheugh | 15 | 31 | 190.73 | 132.73 | 69.87 | 46.02 | 11.39 | 8.63 |
| Isle of May | 48 | 133 | 69.22 | 86.37 | 21.94 | 25.45 | 5.83 | 7.08 |
| Lambay | 14 | 49 | 63.20 | 40.00 | 21.02 | 11.05 | 4.03 | 2.76 |
| Orkney: Copinsay | 29 | 110 | 65.54 | 115.34 | 21.18 | 37.70 | 4.34 | 5.60 |
| Orkney: Muckle Skerry | 47 | 255 | 34.82 | 82.71 | 10.88 | 25.01 | 2.29 | 4.22 |
| Puffin Island | 82 | 458 | 51.46 | 62.77 | 15.33 | 17.89 | 4.14 | 5.44 |
| St Abbs | 15 | 35 | 143.02 | 163.55 | 38.98 | 41.82 | 11.01 | 12.06 |
| Scilly:  St Martins | 34 | 97 | 69.97 | 75.03 | 22.40 | 26.36 | 5.14 | 4.38 |
| Whinnyfold | 20 | 61 | 107.41 | 66.26 | 37.15 | 20.50 | 7.89 | 6.09 |

## Table S3.2 Average Step lengths and turning angles of different kittiwake behavioural states at all colonies.

| **State** | **Mean step length (km)** | **Standard deviation in step length (km)** | **Mean turning angle (μ)** | **Concentration of turning angle (κ)** |
| --- | --- | --- | --- | --- |
| Resting | 0.07 ±0.03 | 0.04 ±0.02 | 0.00 ±0.01 | 10.77 ±10.69 |
| Foraging | 0.23 ±0.10 | 0.26 ±0.10 | 0.03 ±1.19 | 0.34 ±0.20 |
| Transiting | 0.98 ±0.11 | 0.32 ±0.05 | 0.00 ±0.01 | 9.84 ±6.59 |

## Table S3.3 Mean average and standard deviation (SD) of step lengths and mean turning angle and concentration of different kittiwake behaviours classified using a hidden Markov model for each colony.

|  | **Resting** | | | | **Foraging** | | | | **Transiting** | | | |
| --- | --- | --- | --- | --- | --- | --- | --- | --- | --- | --- | --- | --- |
| **Colony** | **Mean step length (km)** | **SD step length (km)** | **Mean turning angle (μ)** | **Concentration of turning angle (κ)** | **Mean step length (km)** | **SD step length (km)** | **Mean turning angle (μ)** | **Concentration of turning angle (κ)** | **Mean step length (km)** | **SD step length (km)** | **Mean turning angle (μ)** | **Concentration of turning angle (κ)** |
| Bardsey | 0.10 | 0.06 | 0.00 | 46.62 | 0.20 | 0.20 | 0.03 | 0.55 | 0.86 | 0.30 | 0.01 | 2.91 |
| Bempton | 0.05 | 0.02 | 0.00 | 15.46 | 0.27 | 0.32 | -0.01 | 0.51 | 1.08 | 0.28 | 0.00 | 21.43 |
| Coquet | 0.05 | 0.03 | 0.03 | 1.11 | 0.41 | 0.33 | -0.03 | 0.47 | 1.06 | 0.27 | 0.00 | 10.85 |
| Colinsay | 0.06 | 0.04 | -0.01 | 7.53 | 0.06 | 0.07 | -3.13 | 0.43 | 0.85 | 0.40 | 0.00 | 2.69 |
| Filey | 0.04 | 0.02 | 0.00 | 6.55 | 0.33 | 0.37 | -0.01 | 0.51 | 1.10 | 0.26 | 0.00 | 23.69 |
| Fowlsheugh | 0.05 | 0.02 | 0.00 | 6.03 | 0.22 | 0.27 | -0.01 | 0.28 | 1.06 | 0.30 | 0.01 | 18.43 |
| Isle of May | 0.04 | 0.02 | 0.00 | 5.19 | 0.18 | 0.21 | 0.02 | 0.14 | 0.95 | 0.33 | 0.00 | 6.13 |
| Lambay | 0.09 | 0.04 | 0.01 | 9.37 | 0.38 | 0.45 | 0.00 | 0.51 | 1.11 | 0.30 | -0.02 | 10.32 |
| Orkney: Copinsay | 0.06 | 0.04 | -0.01 | 4.09 | 0.26 | 0.32 | -0.14 | 0.08 | 1.04 | 0.35 | 0.00 | 7.20 |
| Orkney: Muckle Skerry | 0.14 | 0.11 | 0.00 | 10.84 | 0.22 | 0.29 | 0.68 | 0.01 | 1.02 | 0.34 | 0.00 | 6.46 |
| Puffin Island | 0.07 | 0.04 | 0.00 | 13.98 | 0.23 | 0.25 | 0.01 | 0.34 | 0.87 | 0.33 | 0.00 | 7.54 |
| St Abbs | 0.03 | 0.02 | 0.01 | 4.39 | 0.21 | 0.26 | 0.02 | 0.14 | 0.95 | 0.27 | 0.00 | 9.69 |
| Scilly: St Martins | 0.05 | 0.03 | -0.01 | 9.97 | 0.20 | 0.23 | -0.09 | 0.20 | 1.02 | 0.34 | 0.00 | 10.36 |
| Whinnyfold | 0.07 | 0.04 | 0.00 | 13.22 | 0.17 | 0.21 | -0.03 | 0.26 | 0.99 | 0.32 | 0.00 | 8.23 |

## Table S3.4 Proportion of time spent in each behaviour at each colony.

| **Colony** | **Individuals** | **Resting** | **Foraging** | **Transiting** |
| --- | --- | --- | --- | --- |
| Bardsey | 8 | 0.21 | 0.39 | 0.40 |
| Bempton | 95 | 0.23 | 0.46 | 0.31 |
| Coquet | 36 | 0.42 | 0.33 | 0.25 |
| Colonsay | 79 | 0.17 | 0.55 | 0.28 |
| Filey | 44 | 0.20 | 0.46 | 0.34 |
| Fowlsheugh | 15 | 0.22 | 0.43 | 0.35 |
| Isle of May | 48 | 0.22 | 0.52 | 0.26 |
| Lambay | 14 | 0.29 | 0.43 | 0.28 |
| Orkney: Copinsay | 29 | 0.25 | 0.45 | 0.31 |
| Orkney: Muckle Skerry | 47 | 0.20 | 0.51 | 0.30 |
| Puffin Island | 82 | 0.25 | 0.50 | 0.26 |
| St Abbs | 15 | 0.25 | 0.48 | 0.28 |
| Scilly: St Martins | 34 | 0.19 | 0.55 | 0.27 |
| Whinnyfold | 20 | 0.24 | 0.46 | 0.30 |
|  |  |  |  |  |
| Weighted Mean |  | 0.23 | 0.48 | 0.29 |
| Weighted Standard Dev |  | 0.06 | 0.06 | 0.03 |

Weighted means for Resting, Foraging, and Transiting behaviours were calculated to account for differences in sample sizes across colonies. For each behaviour, the weighted mean (${\bar{\mathcal{X}}}_{\mathfrak{w}})$ was computed as: ${\bar{\mathcal{X}}}_{\mathfrak{w}}= \frac{\sum\left( \mathcal{X}_{\mathcal{i}} \times\mathcal{W}_{\mathcal{i}} \right)}{\sum\mathcal{W}_{\mathcal{i}}}$ where $\mathcal{X}_{\mathcal{i}}$is the mean behaviour value for each colony, and $\mathcal{W}_{\mathcal{i}}$ is the number of individuals in that colony. This approach ensures colonies with larger sample sizes contribute proportionally more to the overall mean. Weighted standard deviations were calculated to reflect variability while accounting for differences in sample sizes. The weighted standard deviation ($\sigma_{\omega})$was computed as: $\sigma_{\omega}=\sqrt{\frac{\sum\mathcal{W}_{\mathcal{i}}\left( \mathcal{X}_{\mathcal{i}}-{\bar{\mathcal{X}}}_{\mathcal{w}} \right)^{2}}{\sum\mathcal{W}_{\mathcal{i}}}}$ where ${\bar{\mathcal{X}}}_{\mathfrak{w}}$ is the weighted mean. This method prevents colonies with smaller sample sizes from disproportionately influencing variability estimates.

# Appendix 4. Kernel Density Estimate Results

## Table S4.1 Percentage of kernel density estimates of separated behaviours captured by “all behaviour” kernel density estimates.

| **Colony** | **Behaviour** | **Mean proportion of behaviour captured** | **Standard deviation** | **Number of Individuals** |
| --- | --- | --- | --- | --- |
| Bardsey | Resting | 0.72 | 0.27 | 8 |
| Bardsey | Foraging | 0.85 | 0.11 | 8 |
| Bardsey | Transiting | 0.60 | 0.18 | 8 |
| Bempton | Resting | 0.96 | 0.09 | 95 |
| Bempton | Foraging | 0.93 | 0.09 | 95 |
| Bempton | Transiting | 0.33 | 0.20 | 95 |
| Coquet | Resting | 0.95 | 0.07 | 29 |
| Coquet | Foraging | 0.85 | 0.13 | 29 |
| Coquet | Transiting | 0.35 | 0.21 | 29 |
| Colonsay | Resting | 0.69 | 0.31 | 36 |
| Colonsay | Foraging | 0.95 | 0.08 | 36 |
| Colonsay | Transiting | 0.43 | 0.20 | 36 |
| Filey | Resting | 0.97 | 0.06 | 79 |
| Filey | Foraging | 0.96 | 0.08 | 79 |
| Filey | Transiting | 0.29 | 0.10 | 79 |
| Fowlsheugh | Resting | 0.97 | 0.06 | 44 |
| Fowlsheugh | Foraging | 0.95 | 0.11 | 44 |
| Fowlsheugh | Transiting | 0.36 | 0.18 | 44 |
| Isle of May | Resting | 0.90 | 0.17 | 15 |
| Isle of May | Foraging | 0.92 | 0.08 | 15 |
| Isle of May | Transiting | 0.41 | 0.25 | 15 |
| Lambay | Resting | 0.91 | 0.09 | 48 |
| Lambay | Foraging | 0.85 | 0.12 | 48 |
| Lambay | Transiting | 0.45 | 0.24 | 48 |
| Orkney: Copinsay | Resting | 0.86 | 0.20 | 14 |
| Orkney: Copinsay | Foraging | 0.92 | 0.10 | 14 |
| Orkney: Copinsay | Transiting | 0.39 | 0.26 | 14 |
| Orkney: Muckle Skerry | Resting | 0.75 | 0.24 | 47 |
| Orkney: Muckle Skerry | Foraging | 0.95 | 0.09 | 47 |
| Orkney: Muckle Skerry | Transiting | 0.39 | 0.19 | 47 |
| Puffin Island | Resting | 0.85 | 0.17 | 82 |
| Puffin Island | Foraging | 0.90 | 0.09 | 82 |
| Puffin Island | Transiting | 0.51 | 0.22 | 82 |
| St Abbs | Resting | 0.96 | 0.08 | 15 |
| St Abbs | Foraging | 0.94 | 0.07 | 15 |
| St Abbs | Transiting | 0.22 | 0.09 | 15 |
| Scilly: St Martins | Resting | 0.88 | 0.16 | 34 |
| Scilly: St Martins | Foraging | 0.91 | 0.13 | 34 |
| Scilly: St Martins | Transiting | 0.49 | 0.24 | 34 |
| Whinnyfold | Resting | 0.91 | 0.12 | 20 |
| Whinnyfold | Foraging | 0.90 | 0.10 | 20 |
| Whinnyfold | Transiting | 0.40 | 0.16 | 20 |

## Table S4.2 Range and weighted grand mean and standard deviations of proportions of separate behaviour kernel density estimates captured within all behaviour kernel density.

| **Behaviour** | **Min** | **Weighted mean** | **Max** | **Weighted SD** |
| --- | --- | --- | --- | --- |
| Sample | 0.90 | 0.92 | 0.94 | 0.01 |
| Resting | 0.80 | 0.87 | 0.97 | 0.10 |
| Foraging | 0.86 | 0.92 | 0.96 | 0.03 |
| Transiting | 0.31 | 0.40 | 0.57 | 0.08 |

Model structure:

model <- glmmTMB(value_prime ~ behaviour + (1| behaviour/Colony),

family = beta_family(link = "logit"), data = beh_vs_all)

## Figure S4.1 Model structure for predicting proportional overlap of the ‘all behaviours’ kernel with the behaviours within it.

We modelled a mixed effects beta regression with a random slope of behaviour nested within colony. (This deals with variation in behaviour at colonies). As we are analysing the proportional overlap of the ‘all behaviours’ kernel with the behaviours within it, they are necessarily non-independent. Because the process we are analysing (the construction of 50% UDs for each behaviour) does not results in complete overlap (e.g. the UD for ‘all behaviour’ is not entirely composed by the UDs of the sub-behaviours) this cannot be more appropriately modelled using, for example, multinomial regression. We have been as cautious as possible in how we interpret this result (including comparison to a random subset), but we do observe that the commuting behaviour is poorly represented by the ‘all behaviours’ kernel and therefore encourage caution when interpreting the p-values.

## Table S4.3 Predicted proportions of each behavioural KDE captured by All Behaviour KDE using utilisation distributions of 50%.

| **Behaviour** | **Predicted** | **95% confidence interval** |
| --- | --- | --- |
| Sample | 0.86 | 0.84, 0.88 |
| Resting | 0.91 | 0.90, 0.93 |
| Foraging | 0.91 | 0.89, 0.92 |
| Transiting | 0.40 | 0.36, 0.45 |

## Table S4.4 Model outputs from mixed effects beta regression comparing the proportion of each behavioural kernel density captured by the total ‘all behaviour’ kernel density using utilisation distributions of 50%. The model intercept shows the estimated proportional overlap in area between ‘sample’ and ‘all behaviour’.

| **Predictors** | **Estimates** | **Confidence intervals** | **P value** |
| --- | --- | --- | --- |
| Intercept: Sample | 6.24 | 5.16 – 7.53 | <0.001 |
| Behaviour: Resting | 1.72 | 1.32 – 2.24 | <0.001 |
| Behaviour: Foraging | 1.63 | 1.25 – 2.12 | <0.001 |
| Behaviour: Transiting | 0.11 | 0.08 – 0.14 | <0.001 |

## Table S4.5 Random effects from mixed effects beta regression comparing the proportion of each behavioural kernel density captured by the total ‘all behaviour’ kernel density using utilisation distributions of 50%.

| **Random Effects** | |
| --- | --- |
| σ^2^ | 0.04 |
| τ00 Colony:Behaviour | 0.09 |
| τ00 Behaviour | 0.00 |
| Ν Colony | 14 |
| Ν Behaviour | 4 |
| Observations | 2225 |

## Following Trevail et al (2019) tracking data in this study were not interpolated. According to MoveHMM package guidance: ‘Small violations of the assumption of regular time intervals and negligible measurement error can be accommodated by moveHMM, e.g., GPS measurement error may be treated as negligible if it is small compared to the scale of movement steps.’ All tracking data were all collected on igotU devices programmed to record every 100 seconds. Overall, mean average time interval for the dataset was 104.3 seconds (standard deviation 9.33).

To investigate the effect of this choice we repeated our analysis to create kernel density estimates based on 50% utilisation distributions using interpolated data. Tracking data was interpolated to 100 seconds using the ltraj function in the package Adehabitat.

## Table S4.6 Predicted proportions of each behavioural KDE captured by All Behaviour KDE using utilisation distributions of 50% after tracking data was interpolated.

| **Behaviour** | **Predicted** | **95% confidence interval** |
| --- | --- | --- |
| Sample | 0.86 | 0.84, 0.88 |
| Resting | 0.93 | 0.92, 0.94 |
| Foraging | 0.92 | 0.90, 0.93 |
| Transiting | 0.41 | 0.37, 0.44 |

## Table S4.7 Model outputs from mixed effects beta regression comparing the proportion of each behavioural kernel density captured by the total ‘all behaviour’ kernel density using utilisation distributions of 50%. The model intercept shows the estimated proportional overlap in area between ‘sample’ and ‘all behaviour’ after tracking data was interpolated.

| **Predictors** | **Estimates** | **Confidence intervals** | **P value** |
| --- | --- | --- | --- |
| Intercept: Sample | 6.26 | 5.26 – 7.45 | <0.001 |
| Behaviour: Resting | 2.07 | 1.62 – 2.64 | <0.001 |
| Behaviour: Foraging | 1.92 | 1.50 – 2.45 | <0.001 |
| Behaviour: Transiting | 0.11 | 0.09 – 0.14 | <0.001 |

## Table S4.8 Random effects from mixed effects beta regression comparing the proportion of each behavioural kernel density captured by the total ‘all behaviour’ kernel density using utilisation distributions of 50% after tracking data was interpolated.

| **Random Effects** | |
| --- | --- |
| σ^2^ | 0.03 |
| τ00 Colony:Behaviour | 0.08 |
| τ00 Behaviour | 0.00 |
| Ν Colony | 14 |
| Ν Behaviour | 4 |
| Observations | 2223 |

## Table S4.9 Predicted proportions of each behavioural KDE captured by All Behaviour KDE using utilisation distributions of 75%.

| **Behaviour** | **Predicted** | **95% confidence interval** |
| --- | --- | --- |
| Sample | 0.90 | 0.88, 0.91 |
| Resting | 0.96 | 0.95, 0.96 |
| Foraging | 0.95 | 0.95, 0.96 |
| Transiting | 0.61 | 0.58, 0.64 |

## Table S4.10 Model outputs from mixed effects beta regression comparing the proportion of each behavioural kernel density captured by the total ‘all behaviour’ kernel density using utilisation distributions of 75%. The model intercept shows the estimated proportional overlap in area between ‘sample’ and ‘all behaviour’.

| **Predictors** | **Estimates** | **Confidence intervals** | **P value** |
| --- | --- | --- | --- |
| Intercept: Sample | 8.76 | 7.54 – 10.18 | <0.001 |
| Behaviour: Resting | 2.46 | 1.98 – 3.04 | <0.001 |
| Behaviour: Foraging | 2.33 | 1.88 – 2.89 | <0.001 |
| Behaviour: Transiting | 0.18 | 0.15 – 0.22 | <0.001 |

## Table S4.11 Random effects from mixed effects beta regression comparing the proportion of each behavioural kernel density captured by the total ‘all behaviour’ kernel density using utilisation distributions of 75%.

| **Random Effects** | |
| --- | --- |
| σ^2^ | 0.01 |
| τ00 Colony:Behaviour | 0.05 |
| τ00 Behaviour | 0.00 |
| Ν Colony | 14 |
| Ν Behaviour | 4 |
| Observations | 2226 |

## Table S4.12 Predicted proportions of each behavioural KDE captured by All Behaviour KDE using utilisation distributions of 95%.

| **Behaviour** | **Predicted** | **95% confidence interval** |
| --- | --- | --- |
| Sample | 0.95 | 0.94, 0.96 |
| Resting | 0.97 | 0.97, 0.98 |
| Foraging | 0.97 | 0.96, 0.97 |
| Transiting | 0.86 | 0.85, 0.88 |

## Table S4.13 Model outputs from mixed effects beta regression comparing the proportion of each behavioural kernel density captured by the total ‘all behaviour’ kernel density using utilisation distributions of 95%. The model intercept shows the estimated proportional overlap in area between ‘sample’ and ‘all behaviour’.

| **Predictors** | **Estimates** | **Confidence intervals** | **P value** |
| --- | --- | --- | --- |
| Intercept: Sample | 19.18 | 16.76 – 21.93 | <0.001 |
| Behaviour: Resting | 0.99 | 1.64 – 2.42 | <0.001 |
| Behaviour: Foraging | 1.54 | 1.27 – 1.87 | <0.001 |
| Behaviour: Transiting | 0.32 | 0.27 – 0.39 | <0.001 |

## Table S4.14 Random effects from mixed effects beta regression comparing the proportion of each behavioural kernel density captured by the total ‘all behaviour’ kernel density using utilisation distributions of 95%.

| **Random Effects** | |
| --- | --- |
| σ^2^ | 0.00 |
| τ00 Colony:Behaviour | 0.05 |
| τ00 Behaviour | 0.00 |
| Ν Colony | 14 |
| Ν Behaviour | 4 |
| Observations | 2226 |

Figure 4.2 Model outputs of proportion of behavioural KDEs captured by All Behaviour KDE using a 50% utilisation distribution. A: Estimated values for proportion of overlap, B: predicted values, C: Random effects. Behaviour 1 and the Intercept are the sub-sample from ‘all behaviour’.

Figure 4.3 Model outputs of proportion of behavioural KDEs captured by All Behaviour KDE using a 75% utilisation distribution. A: Estimated values for proportion of overlap, B: predicted values, C: Random effects. Behaviour 1 and the Intercept are the sub-sample from ‘all behaviour’.

Figure 4.4 Model outputs of proportion of behavioural KDEs captured by All Behaviour KDE using a 95% utilisation distribution. A: Estimated values for proportion of overlap, B: predicted values, C: Random effects. Behaviour 1 and the Intercept are the sub-sample from ‘all behaviour’.

# Appendix 5: Size Estimates of Population-level Key Areas

## Table S5.1 Size of key areas, defined using 50% utilisation distribution, of all behaviour, three separated behaviours (resting, foraging, and transiting) and a sample of all behaviours as small as the smallest behaviour.

| **Colony** | **All behaviour core area (km^2^)** | **Resting core area (km^2^)** | **Foraging core area (km^2^)** | **Transiting core area (km^2^)** | **Sample core area (km^2^)** |
| --- | --- | --- | --- | --- | --- |
| Bempton Cliffs | 546 | 111 | 313 | 1582 | 540 |
| Coquet | 155 | 106 | 150 | 419 | 169 |
| Colonsay | 328 | 71 | 200 | 685 | 293 |
| Isle of May | 500 | 108 | 312 | 710 | 481 |
| Orkney Islands: Copinsay | 189 | 82 | 124 | 470 | 193 |
| Orkney Islands: Muckle Skerry | 181 | 131 | 136 | 270 | 174 |
| Puffin Island | 477 | 170 | 350 | 469 | 479 |
| Scilly: St Martins | 198 | 94 | 137 | 344 | 190 |
| Whinnyfold | 847 | 332 | 524 | 1183 | 807 |
| Mean | 380 | 133 | 249 | 681 | 369 |

lme1 = lmer(log10(area_km) ~ behaviour + (1|colony), data = all_results_df_long)

## Figure S5.1 Model structure for linear mixed effect model to compare the spatial extent (in km^2^) of key area estimates based on a 50% utilisation distribution and calculated using ‘all behaviour’ track data, separated behaviour track data and a control ‘sample’. We included ‘colony’ as a random effect and area estimates were logged.

## Table S5.2 Model outputs from linear mixed effects model comparing the spatial extent of delineated key areas 50% utilisation distribution. Site: Sample is a random subsample of all behaviour.

| **Predictors** | **Estimates** | **Confidence intervals** | **P value** |
| --- | --- | --- | --- |
| **Intercept** | 2.30 | 2.05 – 2.55 | <0.001 |
| **Site: Sample** | -0.02 | -0.19 – 0.15 | 0.830 |
| **Site: Resting** | -0.48 | -0.65 - -0.31 | <0.001 |
| **Site: Foraging** | -0.16 | -0.33 - -0.00 | 0.055 |
| **Site: Transiting** | 0.28 | 0.12 – 0.45 | 0.001 |

## Table S5.3 Random effects from linear mixed effects model comparing the spatial extent of delineated key areas based on a 50% utilisation distribution.

| **Random Effects** | |
| --- | --- |
| **σ^2^** | 0.03 |
| **τ00 Colony** | 0.11 |
| **ICC** | 0.78 |
| **Ν Colony** | 9 |
| **Observations** | 45 |
| **Marginal R^2^ / Conditional R^2^** | 0.314 / 0.846 |

## Figure 5.2 Predicted spatial extent of population-level key areas based on a 50% utilisation distribution from linear mixed effects model. All Behaviour includes all tracking data, ‘Sample’ is a random subsample of the tracking data, Foraging, Resting and Transiting behaviours were defined from tracking data using hidden Markov models. Natural log was removed for the predictive values.

## Table S5.4 Size of key areas defined using 75% kernel density estimates of all behaviour, three separated behaviours (resting, foraging, and transiting) and a sample of all behaviours as small as the smallest behaviour.

| **Colony** | **All behaviour core area (km^2^)** | **Resting core area (km^2^)** | **Foraging core area (km^2^)** | **Transiting core area (km^2^)** | **Sample core area (km^2^)** |
| --- | --- | --- | --- | --- | --- |
| Bempton Cliffs | 1970 | 352 | 861 | 3644 | 1860 |
| Coquet | 255 | 161 | 265 | 524 | 241 |
| Colonsay | 894 | 483 | 482 | 1616 | 855 |
| Isle of May | 893 | 303 | 565 | 1089 | 786 |
| Orkney Islands: Copinsay | 285 | 91 | 124 | 549 | 226 |
| Orkney Islands: Muckle Skerry | 179 | 125 | 99 | 217 | 146 |
| Puffin Island | 733 | 408 | 573 | 710 | 702 |
| Scilly: St Martins | 356 | 145 | 223 | 723 | 336 |
| Whinnyfold | 2007 | 1163 | 1585 | 2127 | 2009 |
| Mean | 841 | 359 | 531 | 1244 | 796 |

## Table S5.5 Model outputs from linear mixed effects model comparing the spatial extent of delineated key areas based on 75% utilisation distribution. Site: Sample is a random subsample of all behaviour.

| **Predictors** | **Estimates** | **Confidence intervals** | **P value** |
| --- | --- | --- | --- |
| **Intercept** | 2.78 | 2.52 – 3.04 | <0.001 |
| **Site: Sample** | -0.04 | -0.15 – 0.07 | 0.473 |
| **Site: Resting** | -0.36 | -0.47 - -0.25 | <0.001 |
| **Site: Foraging** | -0.20 | -0.32 - -0.09 | 0.001 |
| **Site: Transiting** | 0.18 | 0.07 – 0.29 | 0.002 |

## Table S5.6 Random effects from linear mixed effects model comparing the spatial extent of delineated key areas based on a 75% utilisation distribution.

| **Random Effects** | |
| --- | --- |
| **σ^2^** | 0.01 |
| **τ00 Colony** | 0.13 |
| **ICC** | 0.91 |
| **Ν Colony** | 9 |
| **Observations** | 45 |
| **Marginal R^2^ / Conditional R^2^** | 0.191 / 0.925 |

## Figure 5.3 Violin plots of the spatial extent of population-level key area estimates, based on a 75% utilisation, for ‘all behaviour’, a random sample of ‘all behaviour’, and tracking data separated into resting, foraging, and transiting behavioural states. Each circle within the violins represents a different colony, box and whiskers show the overall mean and interquartile range.

## Table S5.7 Size of key areas defined using 95% kernel density estimates of all behaviour, three separated behaviours (resting, foraging, and transiting) and a sample of all behaviours as small as the smallest behaviour.

| **Colony** | **All behaviour core area (km^2^)** | **Resting core area (km^2^)** | **Foraging core area (km^2^)** | **Transiting core area (km^2^)** | **Sample core area (km^2^)** |
| --- | --- | --- | --- | --- | --- |
| Bempton Cliffs | 6318 | 1497 | 3339 | 6324 | 5970 |
| Coquet | 1040 | 500 | 740 | 1304 | 925 |
| Colonsay | 2811 | 1608 | 1750 | 3232 | 2704 |
| Isle of May | 1838 | 1139 | 1484 | 1951 | 1780 |
| Orkney Islands: Copinsay | 1089 | 246 | 376 | 1228 | 946 |
| Orkney Islands: Muckle Skerry | 424 | 257 | 225 | 452 | 347 |
| Puffin Island | 1397 | 1003 | 1148 | 1335 | 1368 |
| Scilly: St Martins | 1752 | 299 | 407 | 1975 | 1465 |
| Whinnyfold | 3848 | 2712 | 3499 | 3639 | 3772 |
| Mean | 2280 | 1029 | 1441 | 2382 | 2142 |

## Table S5.8 Model outputs from linear mixed effects model comparing the spatial extent of delineated key areas 95% utilisation distribution. Site: Sample is a random subsample of all behaviour.

| **Predictors** | **Estimates** | **Confidence intervals** | **P value** |
| --- | --- | --- | --- |
| **Intercept** | 3.24 | 2.99 – 3.49 | <0.001 |
| **Site: Sample** | -0.04 | -0.16 – 0.08 | 0.510 |
| **Site: Resting** | -0.37 | -0.49 - -0.25 | <0.001 |
| **Site: Foraging** | -0.25 | -0.37 - -0.13 | <0.001 |
| **Site: Transiting** | 0.03 | 0.12 – 0.50 | 0.606 |

## Table S5.9 Random effects from linear mixed effects model comparing the spatial extent of delineated key areas based on a 95% utilisation distribution.

| **Random Effects** | |
| --- | --- |
| **σ^2^** | 0.02 |
| **τ00 Colony** | 0.12 |
| **ICC** | 0.89 |
| **Ν Colony** | 9 |
| **Observations** | 45 |
| **Marginal R^2^ / Conditional R^2^** | 0.152 / 0.906 |

## Figure 5.4 Violin plots of the spatial extent of population-level key area estimates, based on a 95% utilisation, for ‘all behaviour’, a random sample of ‘all behaviour’, and tracking data separated into resting, foraging, and transiting behavioural states. Each circle within the violins represents a different colony, box and whiskers show the overall mean and interquartile range.
